# Hypothesis of a potential BrainBiota and its relation to CNS autoimmune inflammation

**DOI:** 10.3389/fimmu.2022.1043579

**Published:** 2022-12-02

**Authors:** Maria L. Elkjaer, Lukas Simon, Tobias Frisch, Lisa-Marie Bente, Tim Kacprowski, Mads Thomassen, Richard Reynolds, Jan Baumbach, Richard Röttger, Zsolt Illes

**Affiliations:** ^1^ Department of Neurology, Odense University Hospital, Odense, Denmark; ^2^ BRIDGE, Department of Clinical Research, University of Southern Denmark, Odense, Denmark; ^3^ Institute of Molecular Medicine, University of Southern Denmark, Odense, Denmark; ^4^ Center for Precision Health, School of Biomedical Informatics, The University of Texas Health Science Center at Houston, Houston, TX, United States; ^5^ Department of Mathematics and Computer Science, University of Southern Denmark, Odense, Denmark; ^6^ Division Data Science in Biomedicine, Peter L. Reichertz Institute for Medical Informatics, Technische Universität Braunschweig and Hannover Medical School, Braunschweig, Germany; ^7^ Braunschweig Integrated Centre of Systems Biology (BRICS), Technische Universität Braunschweig, Braunchweig, Germany; ^8^ Research Unit of Human Genetics, Department of Clinical Research, University of Southern Denmark, Odense, Denmark; ^9^ Department of Brain Sciences, Imperial College, London, United Kingdom; ^10^ Centre for Molecular Neuropathology, LKC School of Medicine, Nanyang Technological University, Singapore, Singapore; ^11^ Chair of Computational Systems Biology, University of Hamburg, Hamburg, Germany

**Keywords:** gut-brain axis, microbiome, microbiota, brainbiome, brainbiota, bacterial transcripts, CNS autoimmunity, smoldering MS

## Abstract

Infectious agents have been long considered to play a role in the pathogenesis of neurological diseases as part of the interaction between genetic susceptibility and the environment. The role of bacteria in CNS autoimmunity has also been highlighted by changes in the diversity of gut microbiota in patients with neurological diseases such as Parkinson’s disease, Alzheimer disease and multiple sclerosis, emphasizing the role of the gut-brain axis. We discuss the hypothesis of a brain microbiota, the BrainBiota: bacteria living in symbiosis with brain cells. Existence of various bacteria in the human brain is suggested by morphological evidence, presence of bacterial proteins, metabolites, transcripts and mucosal-associated invariant T cells. Based on our data, we discuss the hypothesis that these bacteria are an integral part of brain development and immune tolerance as well as directly linked to the gut microbiome. We further suggest that changes of the BrainBiota during brain diseases may be the consequence or cause of the chronic inflammation similarly to the gut microbiota.

## Pathogens and CNS autoimmunity

In the inflammatory demyelinating neurodegenerative disease, multiple sclerosis (MS), autoimmune mechanisms are considered to play a role. Yet, causative autoantibodies or specific target antigens have not been confirmed, and MS lesions are not always associated with infiltrating lymphocytes (1). In addition to the genetic immune components associated with MS (2) the geographic distribution, prevalence, and migration studies support the notion that the exposome including different environmental pathogens is critical for disease onset and course (3). Therefore, there is a possibility that pathogens initiate or maintain the chronic brain damage in MS (4, 5). So far, the focus has mostly been on viruses.

Viruses have long been considered as infectious triggers in MS pathogenesis, as they can alter systemic immune responses and maintain local inflammation. Presence of Epstein-Barr virus (EBV) persisting in the MS brain and in leptomeningeal B cells is controversial (6, 7) Nevertheless, EBV has the strongest association with MS, the risk of which is increased 32-fold after EBV infection (6, 7). Antibody responses generated against the EBV protein EBNA1 outside of the CNS and cross-reacting with GlialCAM within the CNS can be a potential mechanism of cross-reactive autoimmunity (8).

Besides exogenous viruses, human endogenous retroviruses (HERV) have also attained attention in MS. HERVs are ancient retroviruses that have been incorporated into the human genome over millions of years and contribute to both immune tolerance and autoimmunity (9–11). A recent phase 2 clinical trial using IgG4 monoclonal antibody targeting HERV-W env (CHANGE-MS) resulted in decreased loss of cortical and thalamic brain volume (12). An interplay between exogenous viruses and HERV has also been suggested. This dual virus hypothesis of MS proposes transactivation of HERVs by EBV or other pathogens as a trigger, and the translated HERV proteins induce or contribute to chronic inflammation (13).

Besides the viruses in the pathogenesis, the gut microbiota has also been linked to CNS disorders (14). Among physiological conditions, probiotic microorganisms shape both the local and systemic immune responses in the host and regulate autoimmunity (15–17). Gut microbes communicate with the brain through a variety of routes including the vagus nerve, short chain fatty acids, cytokines, and tryptophan levels (18). The gut-brain axis plays a critical role in orchestrating brain development and behavior in humans by modulating functions of microglia and neurogenesis (19). In experimental models, gut microbes participate in maintaining blood-brain barrier (BBB) permeability, influence expression of myelinating genes (20, 21), activation of microglia (22), and limit astrocyte pathogenicity (23). In contrast, a dysbiotic gut microbiome contributes to autoimmune and neurodegenerative diseases (24, 25). In most CNS diseases, the diversity of the intestinal microbiota is markedly reduced. Such reduced diversity also characterizes MS with specific absence of certain stains e.g., *Clostridia* subclusters and *Bacteroides* species (26–28). Obesity, an emerging risk factor for MS, also associates with altered gut microbiome (29, 30).

These studies suggest an indirect role of bacteria regulating CNS immune responses, and particularly, the importance of microbiota in MS pathogenesis. Only a few studies have focused on the direct presence and potential role of bacteria within the brain (31, 32).

## Is there a BrainBiota?

As we discussed, evidence indicate bidirectional microbiota-gut-brain communication, and the important role of microbiota in immune tolerance and autoimmunity (33). This is primarily an indirect regulation of CNS immune responses by the gut microbiota (34). The presence of bacteria in the normal brain has also been investigated. The discovery of bacteria near brain vessels without inflammation and damage (35) may support our hypothesis of the BrainBiota. Previous works has suggested the origin of brain bacteria from the blood by a low-level breaching of the BBB balanced by active removal (31, 32).

### Evidence of bacteria in the brain of CNS diseases

Morphological evidence of bacterial LPS and K99 pili protein along with *E. coli* DNA were found in brains of Alzheimer disease (AD) patients and age-matched controls (36). LPS was colocalized with Aβ deposits, and levels of the K99 pili protein and LPS were greater in AD compared to control brains. The authors speculated that *E. coli* molecules in the AD and control brains might originate from the blood and be carried by monocytes or cytotoxic T cells or through the disrupted BBB (36).

Additionally, 173 different bacterial- and phage-derived sequences were detected in normal and abnormal brains of HIV/AIDS patients compared to other non-disease and non-neurological disease controls (37). Bacterial rRNA quantities of the α-proteobacteria were similar regardless of underlying immune status (37). The authors concluded that brain bacteria do not appear to be derived from the predominant populations at other human body sites and may be transported to the brain as intracellular agents.

In the frontal lobe and striatum of Huntington’s disease (HD) patients, bacterial reads of *Pseudomonas, Acinetobacter*, and *Burkholderia* were discovered with 16S rRNA-seq and validated with qPCR (38). The authors argued that specific microbiota might progressively colonize the HD brain and excrete extracellular enzymes and toxic compounds.

In MS brain tissue, bacterial peptidoglycan (PG) was present in a higher quantity in macrophages and dendritic cells compared to control brain tissue. The authors discussed that redistribution of PG by antigen presenting cells from mucosal surfaces to the brain may elicit CNS inflammation in the absence of bacteria and can be a microbial mediator in sterile inflammation (39). The tissue resident CD8^+^ cells in the parenchyma also support the pathogen-related pathogenesis (1, 40, 41). The possibility that bacteria or bacterial antigens may be present in MS brains have also been indicated by detecting mucosal-associated invariant T cells (MAIT) in MS lesions, and the expression of MR1 that presents antigens for MAIT cells (42, 43). MAIT cells may be programmed to respond when they are exposed to bacterial antigens together with inflammatory signals to avoid unwanted tissue inflammation (44). Microbial metabolites of vitamin B9 and especially vitamin B2 (riboflavin) derivatives bind to MR1 and can activate MAIT cells (45, 46). The number of MAIT cells is reduced in germ-free mice (47) and the gut microbiota modulate the development of MAIT cells (47, 48).

### Evidence of bacteria in the healthy brain and comparison to MS and other brain diseases

RNA sequencing data from 73 macrodissected white matter (WM) areas of 10 patients with progressive MS and 25 control WM areas from five cases with non-neurological disease were extracted from our previous studies (11, 49–51). The MS brain areas included normal-appearing WM (NAWM), active lesions with influx of systemic inflammatory cells, remyelinating lesions with partial repair, inactive lesions with little to no inflammatory activity, and chronically active (slowly expanding) lesions with glial responses at the edge (52–55). The non-human-mapping read fraction underwent metafeature classification, and different bacterial microbes were identified with CLARK-S (56) as described in the MetaMap pipeline (57).

**Figure 1:**
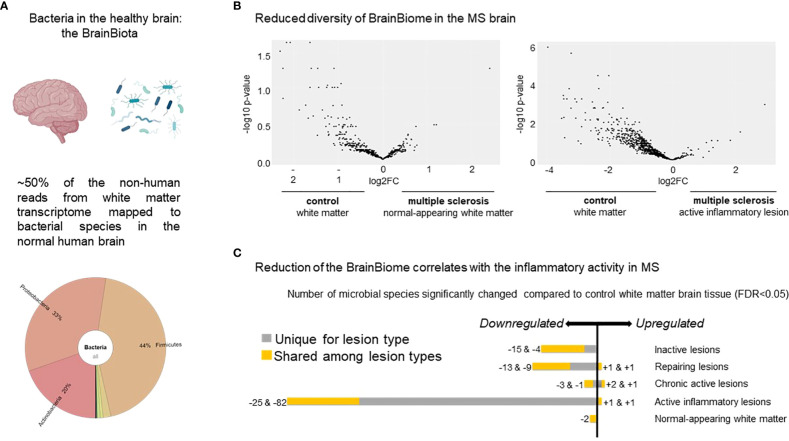
Distribution of microbes based on transcripts in the healthy brain and MS lesions. **(A)** Krona plot visualizing the distribution of the different microbes detected in the healthy brain based on RNA sequencing. The figure highlights the presence of three major bacterial phyla. **(B)** Volcano plot of the distribution of the different microbes represented by the dots based on differential expressed genes in NAWM *vs* control WM (n=90 transcripts) (left panel) and in active lesion type *vs* control WM (n=294 transcripts) (right panel). The figure indicates reduced diversity in both the MS NAWM and active lesions with inflammatory activity based on the higher expressed transcripts from different microbes in control WM. **(C)** Number of microbes significantly changed in the different MS white matter lesion types compared to control WM. Note the most reduced diversity in the active lesions with the highest inflammatory activity. The bacteria and brain are created by Biorender.com.

All five control cases had an enriched microbiome present in the WM tissue, although the cause of death was non-infectious diseases, such as ovarian, renal, tongue or colon cancer and heart failure (**Figure 1A**).

When MS WM was compared to control WM, we found significantly fewer bacterial species based on transcripts (**Figure 1B**). In addition, 154 out of 161 identified microbes based on differentially expressed transcripts were significantly reduced in the MS brain compared to control WM (**Figure 1C**). The most pronounced reduction was observed in active lesions based on 294 differentially expressed transcripts from different microbes, suggesting that the diversity may depend on the inflammatory activity of the lesions (**Figures 1B, C**).

In contrast, another study did 16S rRNA gene sequencing on healthy donors and Parkinson’s disease (PD) patient brains to test the hypothesis of both a potential brain microbiome and a potential imbalance in PD, but their data could not support either (58). Most bacterial reads were off-target amplicons, and they highlight the importance of using extensive controls and to be aware of false positive signals from bioinformatic tools.

Our results are also based on low level bacterial read coverage, which may be due to a much lower magnitude of bacterial transcripts from a brain bacteria niche compared to human transcripts. Thus, false positive signals among the identified individual species are expected. Therefore, we focused on the phyla level and the significant differences between inflammation *versus* non-inflammation.

Postmortem contamination is unlikely to influence the overall results in our study: the brains were removed within 8-30 hours and prepared by the same laboratory. Procedures from macrodissection to sequencing were handled by the same person, and diseased and control brains were mixed during the whole process. Therefore, disease-specific (**Figure 1B**) and inflammation-specific differences (**Figure 1C**) cannot stem from varying sample preparation or any other biased contamination. The possibility of bacterial contamination from the blood is low, as we detected downregulation of bacterial transcripts in the majority of the active MS lesions, which are localized near and around blood vessels. There is also very little evidence of BBB damage in progressive MS, and the immune infiltration is an active process and highly selective (59). Therefore, the downregulation of bacterial transcripts may rather be explained by rapid degradation of resident microbes in the active lesion full of phagocytes.

### The difficulty of detecting microbiota in the brain

The lack of focused search might have hindered the detection of bacterial sequences or bacteria in the brain so far. Most sequences in human brain studies were obtained by human-specific probes or non-human reads were discarded during preprocessing. Pathological examination needs electron microscopy with expertise to differentiate bacteria from cellular organelles. Additionally, microbial sequences from human brain tissues are much less abundant than human transcripts. RNA content is about two orders of magnitude lower than that of eukaryotic cells, and bacterial mRNAs are less stable than their eukaryotic counterparts (60). Therefore enrichment prior to sequencing is necessary, e.g., depletion of human rRNA, tRNA and mRNA. Lastly, the diversity of microbes demands larger cohorts for complete alignment of a potential BrainBiome.

## How do bacteria reach the brain?

**Figure 2:**
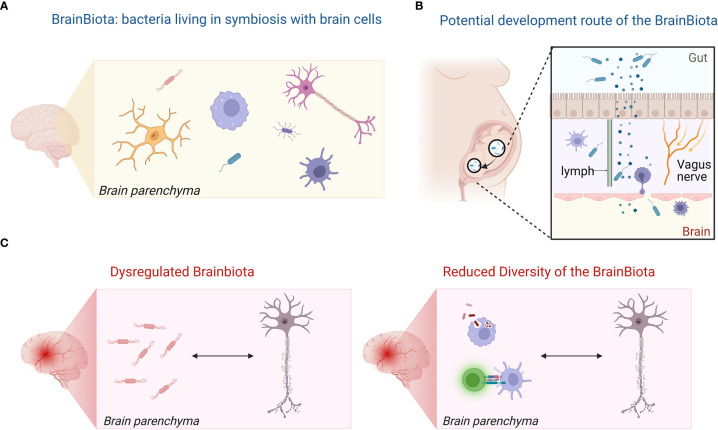
Hypothesis of the BrainBiota and its role in MS. **(A)** The healthy brain has a microbiota (BrainBiota). **(B)** The BrainBiota overlaps with the gut microbiome as it originates from the early gut microbiome. **(C)** Dysregulated BrainBiota may contribute and maintain chronic inflammation in brain diseases or be the result of the inflammatory milieu.Illustration was created in BioRender.

The source of bacterial proteins, metabolites, transcripts, and the bacteria themselves is unclear. While blood-borne origin from a low-level breaching of the BBB has been suggested, we may consider another possibility: the symbiotic colonization of the human brain by bacteria, i.e., the BrainBiota. Accordingly, we hypothesize that the healthy brain may have a microbiota (BrainBiota) (**Figure 2A**), which overlaps with the gut microbiota (**Figure 2B**), and there is a causative link between brain diseases and an imbalanced BrainBiota (**Figure 2C**). Thus, BrainBiota may even have a regulatory function that protects against organ-specific autoimmunity. We postulate that specific, albeit changes in the BrainBiota can the cause or effect of chronic inflammation and contribute to life-long brain disease.

Microorganism communities are spread around the human body in different areas, i.e. in the gut environment (61), the skin (62), the urogenital areas (63, 64), the lungs (65), the eyes (66) and the mouth (67), where they play a crucial role in maintaining health by the perfect balance in types and numbers of various bacteria, fungi, and other microorganisms. Tissue-specific microbial signatures have also been detected in blood, in liver and in distinct types of adipose tissue in type 2 diabetes (68). The main phyla are not consistent across the organs, but they seem to be consistent within the same tissue regardless of condition (68). The three main phyla detected in the brain by our and other studies were major phyla in the gut *(Firmicutes, Actinobacteria, Proteobacteria*) (69). In the early postnatal period, mostly *Proteobacteria* and *Actinobacteria* are dominant in the gut. With time, the gut microbiota becomes more diverse and *Firmicutes* and *Bacteroidetes* emerge (70, 71). Therefore, we may speculate that during fetal development, low-level bacteria could migrate through the open gut-brain axis and live in symbiosis controlled by the CNS immune system similarly to the skin and gut (**Figure 2B**). Another possibility is that the bacteria migrate to the brain through the lymphatic route. Bacterial neurotoxic metabolites have also been detected in the cerebrospinal fluid (CSF) of MS patients correlating with biomarkers of neurodegeneration in MS (72). The authors suggested that those bacterial neurotoxins originated from the gut and traveled to the CSF through gut-brain axis (72).

## What can be the function of BrainBiota and how does it relate to pathology?

A potential BrainBiota may play a role in brain development and maintaining homeostasis, may educate the brain’s immune system, and produce metabolites/neurotransmitters similarly to the role of microbe communities in other organs. We found that the increased immune responses in the active MS lesions correlated with the decreased biodiversity by CLARK-S in our study. Therefore, we hypothesize that in a pathological setting the BrainBiota becomes dysbalanced similarly to the gut microbiota. This can either be the result of abnormal immune responses in the brain, or the tissue injury induces collateral damage towards the BrainBiota, e.g., by direct killing or indirectly through nutrition deficit or oxidative stress. The resultant dysbiotic environment may also promote chronic inflammation in a vicious circle. A primarily dysregulated brain microbiota may also elicit protective immune responses to control the bacterial growth and initiate autoimmunity. Of note, such event may fit well to the “inside out” theory of MS proposing a primary event in the CNS eliciting secondary autoimmune responses and can also explain inflammation primarily driven by microglia in the evolving concept of “smoldering” MS (73). However, the similarity between the control WM and MS NAWM may suggest that a potential imbalance may be the consequence of inflammation rather than a primary cause (**Figure 2C**).

Therefore, we hypothesize that *(i)* the brain has a microbiota (BrainBiota) (**Figure 1A**); *(ii)* it overlaps with the gut microbiota (**Figure 1B**); and *(iii)* there is a direct link between brain disease and altered BrainBiota (**Figure 1C**). Additionally, *(iv)* the BrainBiota may have a regulatory function that protects against organ-specific autoimmunity. Accordingly, we postulate that abnormal change in the BrainBiota may be a potential cause or consequence of chronic inflammation and life-long brain disease.

## Limitations

The existence of a potential BrainBiota and especially its hypothesized regulatory function must be properly studied, and existing results should be confirmed by additional works. The current major limitations are (i) number of studies directly searching for bacteria in the brain; (ii) the limited sample size in studies; (iii) the low bacterial read coverage; (iv) no examination of the same biological material on multiple molecular levels; and (v) examination on species level. Overcoming these obstacles can help to support the hypothesis of BrainBiota and determine the composition of the bacterial species and their functions. The limited number of studies that directly examined bacteria in the brain, their major limitations and the interpretation of the results are summarized in **Table 1**.

**Table 1:** Examination of bacteria in the human brain.

Study	Methods	Findings	Interpretations	Limitations
Schrijver et al. (2001) (38)	Immunohistochemistry of bacterial peptidoglycan (PG) on brain samples from 17 MS patients (n=17) and controls (n=10).	The amount of PG in antigen presenting cells were higher in MS brains compared to control brains. Those PG-containing cells were mainly at the active lesion edge or around blood vessels.	Pathogenic role: PG-containing cells contribute to local immunoreactivity. Origin: from outside of the CNS by PG-containing cells.	No validation of antibody specificity (positive/negative controls) or examination of other bacterial proteins or transcripts.
Zhan et al. (2016) (35)	Western blots, immunocytochemistry, and *E coli* DNA sequencing in AD patients (n=24) and controls (n=18).	Gram-negative bacterial LPS and E. coli K99 pili were detected in both AD and control brains with all three methods. The specific *E coli* protein and DNA increased at injury with parallel increase of IL1β and granzyme B.	Pathogenic role: associated with AD pathology. Origin: from outside of the CNS (i) by the gut-brain-axis, (ii) carried by cytotoxic T cells, NK cells, or (iii) by urinary tract or other infections.	Limited to only *E coli*.
Branton et al. (2013) (36)	RNA and bacterial 16s rRNA sequencing in brains from HIV patients (n=12), other disease controls (ODC) (n=14) and surgical from epilepsy (SURG) (n=6). Additional *in situ* hybridization and *in vivo* cerebral implantation of human bran into mice.	Consistent presence of bacterial rRNA and associated bacterial products in the human brain. *Proteobacteria* was the most frequent class in all human brain samples with similar quantities in HIV and ODC. However, it was reduced in SURG.	Unexplained role: similar bacterial rRNA quantities in HIV and ODC groups despite increased host neuroimmune responses in the HIV group. Origin: oral consumption or inhalation with eventual transport to the brain as intracellular agents in activated leukocytes trafficking into the brain.	Limited to bacterial class level and lack of validation of *Proteobacteria.* Potential contamination of autopsy brains as less classes were detected in the surgical brain samples.
Roberts et al. (2018) (32)	Serial section analysis of ultrastructural of noninfectious or nontraumatic human postmortem brains (n=34).	Bacteria were detected in different amounts without signs of inflammation. In germ free mouse brains with identical procedure, there were no bacteria eliminating contamination.	Unexplained role: specific location, deep within specimens without sign of inflammation Origin: unclear	No genomic, transcriptomic, or targeted sequencing to confirm bacteria. Lack of details and results, as all data has not been published yet.
Alonso et al. (2019) (37)	16s rRNA sequencing in HD patients.	Multiple bacteria as *Pseudomonas*, *Acinetobacter*, and *Burkholderia* were detected in the frontal cortex and striatum in HD brains.	Pathogenic role: risk factor, induction of general brain atrophy and contribution to time of appearance of symptoms. Origin: specific bacteria may progressively infect and colonize at susceptible areas in brains of HD patients.	Lack of non-neurological disease brain samples and validation on other molecular levels.
Bedarf et al. (2021) (57)	16s rRNA sequencing of healthy brains (n=22) and PD brains (n=25) as well as pathogen-free mouse brains (n=5) and germ-free mouse brains (n=3).	No va lid taxonomic bacterial signals for infection in PD brains or for residing in healthy human brains.	No brain microbiome or bacterial infection of the brain.	No validation of the false-positives with other methods or comparative methods for removal of off-target amplicons. Limited to 16s rRNA, while the conclusion could have been stronger if metagenomics were included.
Our results	RNA sequencing of different brain lesions of MS patients (n=10) and brains from non-neurological disease (n=5)	Increased bacterial reads in control tissue compared to MS tissue. The amount of bacterial diversity decreased with increased inflammation at tissue site.	Regulatory role: Direct relation to early postnatal gut bacteria. Part of brain development and maintaining immunotolerance in a symbiotic environment co-existing with brain cells (a BrainBiota). Origin: Early colonization of the brain from the gut (intrauterine)?	No validation on other molecular levels, and read coverage was too low to determine specific bacterial species or functions.

## Conclusion

In summary, transcripts indicate that gut microbes may be present in non-infected human brains, and we hypothesize that they are living in equilibrium in the brain similarly to the gut microbiota (31, 32). We propose that the BrainBiota is related to the early gut microbiota and contributes to brain development. We also hypothesize that a dysbalanced BrainBiota associates with CNS damage and repair by either cause or consequence. In the light of this, the gut-brain axis may contribute to CNS autoimmunity in two ways: the gut microbiota can shape CNS autoimmunity indirectly, while the BrainBiota originating from the gut contributes directly. In MS, the dysbalance of the BrainBiota may also explain chronic inflammation by compartmentalized adaptive and innate immune responses in the evolving concept of “smoldering” MS. Exploration of such theories needs well-designed cohorts as described previously (31). However, if proven, this will open entirely new research fields for autoimmune, primary degenerative, and mental brain disorders with far reaching effects on diagnosis, treatment, and prognosis.

## Data availability statement

Publicly available datasets were analyzed in this study. This data can be found here: https://www.ncbi.nlm.nih.gov/geo/query/acc.cgi?acc=GSE138614.

## Author contributions

ME and ZI contributed to concept and design, obtaining research grants, acquisition, analysis, and interpretation of data, drafting of the manuscript, revision of the manuscript, and critical revision of the manuscript for important intellectual content. LS contributed to analysis of data and revision of manuscript. TF contributed to acquisition and analysis of data, drafting of the manuscript, revision of the manuscript, and statistical analysis. L-MB contributed to analysis of data and revision of the manuscript. TK contributed to analysis of data, drafting of the manuscript, revision of the manuscript, and critical revision of the manuscript for important intellectual content. MT contributed to concept and design, acquisition, analysis of data, and revision of the manuscript. RRe contributed to acquisition of data, analysis and interpretation of data, and revision of the manuscript. JB contributed to concept and design, obtaining research grants, acquisition, analysis, and interpretation of data, drafting of the manuscript, revision of the manuscript, and critical revision of the manuscript for important intellectual content. RRö contributed to analysis and interpretation of data, and revision of the manuscript. All authors contributed to the article and approved the submitted version.

## Funding

MLE is grateful for financial support from Lundbeckfonden (no. R347-2020-2454). ZI is grateful for financial support from Independent Research Fund Denmark (DFF 9039-00370B), Lundbeckfonden (R118-A11472), Scleroseforeningen (A25341, A29926, A31829, A33600), University of Southern Denmark (14/24200), Odense University Hospital (5798002573633). JB is grateful for financial support from the Center for Data and Computing in Natural Sciences (CDCS), and by his VILLUM Young Investigator Grant nr.13154. Furthermore, this project has received funding from the European Union’s Horizon 2020 research and innovation programme under grant agreement No 777111. This publication reflects only the authors’ view and the European Commission is not responsible for any use that may be made of the information it contains.

## Conflict of interest

The authors declare that the research was conducted in the absence of any commercial or financial relationships that could be construed as a potential conflict of interest.

## Publisher’s note

All claims expressed in this article are solely those of the authors and do not necessarily represent those of their affiliated organizations, or those of the publisher, the editors and the reviewers. Any product that may be evaluated in this article, or claim that may be made by its manufacturer, is not guaranteed or endorsed by the publisher.
